# Effects of mini-basketball training program on social communication impairments and regional homogeneity of brain functions in preschool children with autism spectrum disorder

**DOI:** 10.1186/s13102-024-00885-7

**Published:** 2024-04-24

**Authors:** Yang Yang, Dandan Chen, Kelong Cai, Lina Zhu, Yifan Shi, Xiaoxiao Dong, Zhiyuan Sun, Zhiyuan Qiao, Yahui Yang, Weike Zhang, Haiyong Mao, Aiguo Chen

**Affiliations:** 1 College of Physical Education, Yangzhou University, Yangzhou, Jiangsu China; 2 Nanjing Sport Institute, Nanjing, Jiangsu China

**Keywords:** Mini-basketball, Autism spectrum disorder, Preschool children, Social communication impairments, Regional homogeneity, Magnetic resonance imaging

## Abstract

**Background:**

Social communication impairments (SCI) is a core symptom of autism spectrum disorder (ASD) and is marked by challenges in social interaction. Although physical exercise has been shown to improve SCI, this finding has not been supported by comprehensive scientific evidence. Existing research has established a strong link between the SCI in children with ASD and abnormalities in regional homogeneity (ReHo). Therefore, investigating the effects of physical exercise on SCI and Reho in patients with ASD may help to elucidate the neurological mechanisms involved.

**Methods:**

The present study included 30 preschool children diagnosed with ASD, with 15 participants in each group (experimental and control). The experimental group underwent a 12-week mini-basketball training program (MBTP) based on routine behavioral rehabilitation, while the control group only received routine behavioral rehabilitation. The Social Responsiveness Scale-Second Edition (SRS-2) was employed to assess SCI in both groups. Resting-state functional magnetic resonance imaging technology was used to evaluate ReHo in both groups.

**Results:**

After 12-week of MBTP, significant group × time interactions were observed between the experimental and control groups in total SRS-2 scores (*F* = 14.514, *p* < 0.001, *η*_*p*_^*2*^ = 0.341), as well as in the domains of social cognition (*F* = 15.620, *p* < 0.001, *η*_*p*_^*2*^ = 0.358), social communication (*F* = 12.460, *p* < 0.01, *η*_*p*_^*2*^ = 0.308), and autistic mannerisms (*F* = 9.970, *p* < 0.01, *η*_*p*_^*2*^ = 0.263). No statistical difference was found in the scores for the social awareness subscale and social motivation subscale in the group × time interaction (all *p >* 0.05). The experimental group exhibited increased ReHo in the right Cerebellum_Crus1 and right parahippocampal gyrus, coupled with decreased ReHo in the left middle frontal gyrus (orbital part), left superior frontal gyrus (dorsolateral), left postcentral gyrus, and right superior parietal gyrus. Furthermore, a decrease in ReHo in the left postcentral gyrus positively correlated with changes in social communication scores in SCI behaviors (*p* < 0.05).

**Conclusions:**

Our study underscores the effectiveness of a 12-week MBTP in ameliorating SCI and abnormalities in ReHo among preschool children with ASD.

**Trial registration:**

The trial is retrospectively registered on the Chinese Clinical Trial Registry (ChiCTR1900024973; August 5, 2019).

## Background

Autism Spectrum Disorder (ASD) is a neurodevelopmental condition typically diagnosed in early childhood [1, 2]. According to the latest survey by the Centers for Disease Control and Prevention (CDC), the prevalence of ASD among children of age eight is 1 in 36, with an increasing trend [3]. Social communication impairments (SCI) represent a core symptom of ASD, encompassing challenges in both verbal and non-verbal aspects of social interaction [2]. These difficulties expose individuals to heightened risks such as bullying, social isolation, friendship issues, and increased susceptibility to mental health problems [4]. Thus, addressing SCI in children with ASD is crucial. Early intervention before the age of three has shown significant enhancements in cognitive abilities, social skills, and adaptive functioning, emphasizing the potential benefits of interventions for preschool-aged children diagnosed with ASD [5]. Currently, addressing the challenge of effectively alleviating SCI in children with ASD has emerged as a primary focus in ASD rehabilitation training.

Over the years, various intervention methods have been employed to enhance SCI in these children, including pharmacotherapy [6], sensory integration training [7], music therapy [8], pivotal response training [9], and physical exercise training [10]. Research has substantiated the effectiveness of these approaches in ameliorating SCI symptoms. Notably, physical exercise has attracted significant interest from researchers in the fields of medicine and special education due to its advantages, such as promoting teamwork, ease of implementation, and absence of side effects [11, 12]. It has shown significant positive effects in the rehabilitation treatment of individuals with ASD. Previous studies have indicated that physical exercise could positively impact SCI in children with ASD, with interventions like equestrian training [13], swimming [14], Kata [15], and basketball interventions [16]. Previous research highlighted the effectiveness of team sports-based mini-basketball in improving SCI in individuals with ASD [17]. In this respect, Wang et al. [18] conducted a mini-basketball intervention in individuals with ASD, revealing significant improvements in core symptoms and executive function compared to a control group. While existing studies suggest the positive impact of physical activity on SCI in individuals with ASD, many have concentrated on behavior aspects, resulting in a lack of comprehensive scientific evidence to fully substantiate this finding.

Recent advancements in neuroimaging studies have contributed valuable insights by establishing a robust correlation between SCI in individuals with ASD and associated developmental deficits [19]. Utilizing structural magnetic resonance imaging (MRI), research has shown that enhancements in social abilities among children with ASD are intricately tied to improvements in white matter integrity [20]. Task-based functional MRI studies have further affirmed abnormal activation patterns in the brains of individuals with ASD, particularly in social brain regions when performing relevant social tasks [21]. Studies based on resting-state magnetic resonance imaging (rs-fMRI) have shown that the brain network connectivity of visual and sensorimotor regions in individuals with ASD exhibits significant divergence from that observed in neurotypical individuals, characterized by heightened functional connectivity [22]. In a study by Yang et al. [23], rs-fMRI was employed to examine the effects of physical exercise regimen on SCI and the functional connectivity of brain networks in ASD patients. The findings revealed substantial improvements in both SCI and the executive control network, with a discernible correlation between the two. While these studies have unveiled the neural mechanisms of SCI in individuals with ASD, emphasizing white matter volume, functional connectivity of brain networks, and activation patterns, it should be acknowledged that neuroimaging data analysis methods centered on long-range interregional temporal correlations might not fully capture local synchrony within specific brain regions.

Regional homogeneity (ReHo) is an effective way to measure the synchronization of functional activity in nearby brain regions, providing insight into the synchronization of local neural activity [24]. Compared to traditional MRI analysis methods, it is less susceptible to external stimuli and has high retest reliability, making it a feasible bio-marker for neuropsychiatric disease research [25]. The ReHo analysis method has been extensively applied in various populations, including those with attention-deficit/hyperactivity disorder (ADHD) [26], depression [27], schizophrenia [28], amongst others. Cross-sectional research on individuals with ASD has shown that individuals with ASD exhibit higher ReHo values in the cerebellum and left superior temporal gyrus when compared to typically developing (TD) children. Conversely, patients with ASD show decreased ReHo values in frontal and parietal regions. Additionally, the ReHo values in the parietal regions of individuals with ASD were positively correlated with SCI [29, 30]. These preliminary studies suggest a potential association between specific brain regions exhibiting abnormal ReHo and the development of ASD. Interestingly, research also suggests that regular physical exercise can enhance children's behavioral performance by modulating ReHo values in the brain. For example, Chen et al. [31] conducted a study administering a 30-minute moderate-intensity aerobic exercise intervention to 10 TD children, revealing that improvements in executive function were associated with enhanced resting-state ReHo in brain function. Similarly, a study of patients with ADHD showed that an equine-assisted therapy intervention led to symptom improvement, which was linked to a decrease in ReHo in the precuneus cluster on the right side [32]. However, longitudinal intervention studies for the ASD population with ReHo deficiencies are limited, necessitating further research to substantiate these hypotheses.

Based on the aforementioned findings, a robust association between physical exercise and improvements in SCI among children with ASD is evident. However, it should be borne in mind that most studies have predominantly concentrated on behavior aspects, thereby lacking comprehensive scientific evidence to unequivocally support this perspective. Prior research has established the correlation between SCI in individuals with ASD and the integrity of white matter as well as functional connectivity within brain networks. Yet, these studies fall short of fully characterizing the local synchronization of voxels in specific brain regions. The application of ReHo emerges as a valuable approach for assessing the synchronization of functional activity in nearby brain regions. Despite this, there is a limitation in longitudinal intervention studies for the ASD population with ReHo deficiencies. Further exploration of the relationship between a mini-basketball training program (MBTP) and ReHo, as well as the connection between SCI and ReHo, would enrich our understanding of how physical exercise can enhance SCI in individuals with ASD at the neural level. Consequently, two hypotheses were formulated for investigation: 1) A 12-week MBTP has the potential to improve both SCI and ReHo in patients with ASD; 2) Mini-basketball holds promise in enhancing SCI in patients with ASD by modulating their ReHo.

## Methods

### Participants

**Fig. 1:**
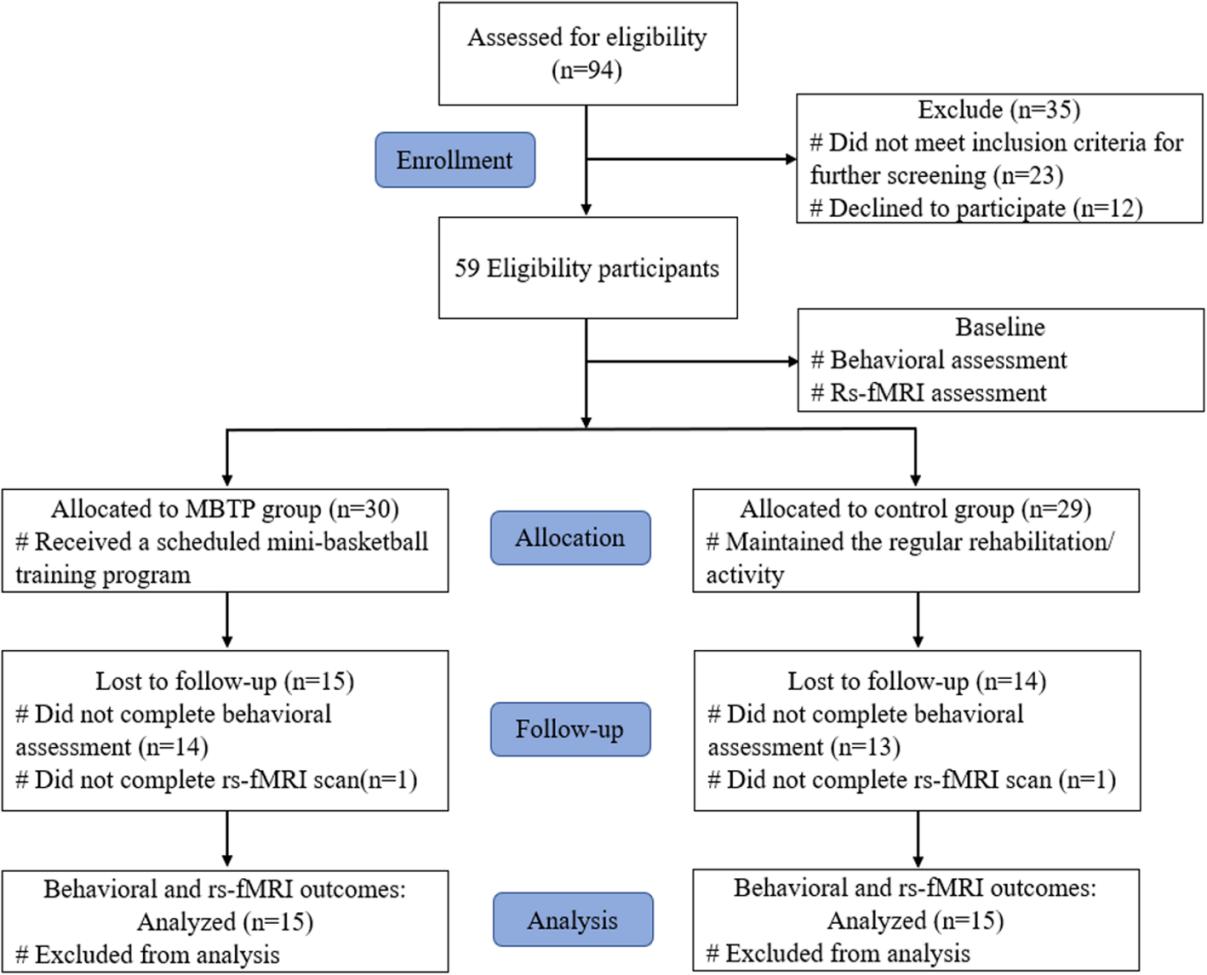
CONSORT flowchart of study participants

Ninety-four children were recruited from two special education centers in Yangzhou City, with inclusion criteria as follows: (1) Han nationality, (2) aged between 3-6, with moderate to severe ASD as diagnosed according to the DSM-V, (3) meeting specific MRI scanning requirements, including the absence of any metal implanted devices (such as teeth fillings), electronics, magnetics, or mechanics (such as cardiac pacing devices), and (4) written informed consent provided by the participants' guardians. Exclusion criteria comprised: (1) engagement in any basketball-related activity in the last six months, (2) presence of neurological or mental conditions such as epilepsy and parkinsonism, (3) physical disability, hearing or vision impairment, (4) individuals with a previous head injury, and (5) use of drugs impacting the central nervous system within the last six months. Thirty-five children were excluded based on these criteria. This quasi-experimental study randomly assigned participants from one institution to the experimental group and participants from another institution to the control group. The experimental group consisted of 30 participants, while the control group comprised 29 participants. Fourteen participants in the experimental group failed to complete the posttest behavioral assessment, and one participant failed to complete the posttest rs-fMRI scan. In the control group, 13 participants failed to complete the posttest behavioral assessment, and one participant failed to complete the posttest rs-fMRI scan, resulting in a total loss of 29 participants. Ultimately, 30 preschool-aged children with ASD were enrolled in this study (Fig. 1). Given the significant participant attrition that could potentially compromise the subsequent statistical analyses, we utilized G*Power software to calculate the required sample size [33]. The parameter settings for this calculation were as follows: statistical test = ANOVA for repeated measures, within-between interaction; effect size f = 0.30; statistical power = 0.80; significance level (α err prob) = 0.05; correlation among repeated measures = 0.5; and number of groups = 2; number of measurements = 2. The analysis revealed that 24 subjects in total, with 12 in each group (experimental and control), would adequately fulfill the statistical analysis prerequisites. Consequently, the number of participants met the requirements for statistical analysis in this study.

### Study design

The present study employed a 2 × 2 mixed experimental design, where group (experimental and control) served as the between-subjects factor, and time (baseline and posttest) served as the within-subjects factor. Ethical approval was obtained from the Ethics and Human Protection Committee of the Affiliated Hospital of Yangzhou University, and the study was registered with the Chinese Clinical Trial Registry (ChiCTR1900024973). Informed consent was obtained from the parents or guardians of all participants.

The experiment consisted of three segments: a baseline, an MBTP, and a posttest. We conducted baseline data collection before the start of the experiment and follow-up data collection immediately after the completion of the 12-week MBTP. Consistency in terms of location, personnel, and testing tools was maintained throughout the process. All participants underwent a standardized Applied Behavior Analysis (ABA) rehabilitation program. The experimental group additionally underwent the MBTP, while the control group maintained their regular study and daily routine, refraining from engaging in similar physical activities.

### Mini-basketball training program

**Fig. 2:**
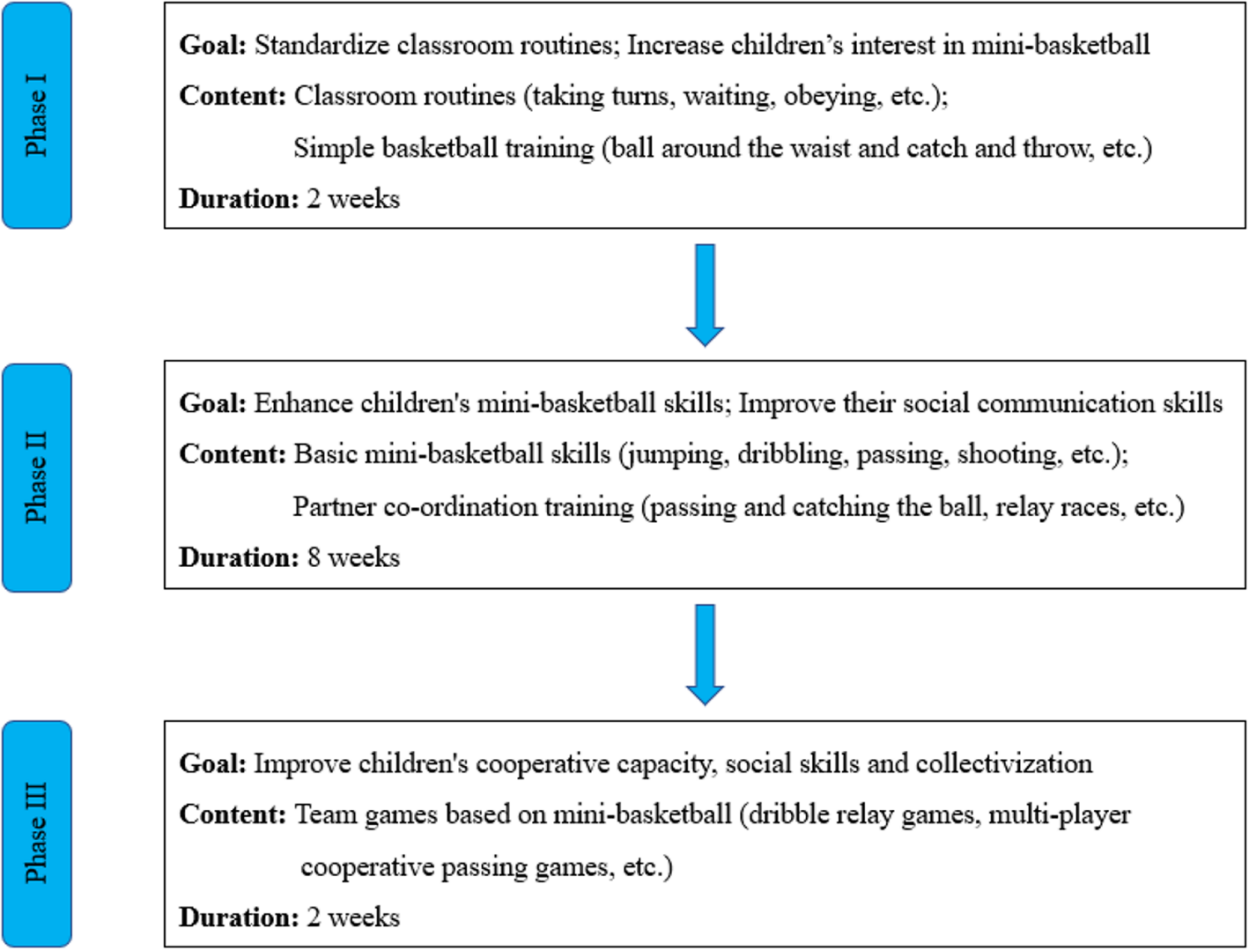
Mini-basketball Training Program

Mini-basketball is a sport designed for children that involves using a smaller-sized basketball. It retains the characteristics of regular basketball while accommodating the physical traits of children, such as their smaller stature, hand size, and limited strength. This promotes the comprehensive development of children's physical fitness effectively. In addition, during the implementation of the mini-basketball intervention program, each child with ASD was accompanied by a guardian who helped them complete ball handling exercises, passing, and dribbling drills. The head coach is a professional in physical education, and every session was supported by teaching assistants who collaborated with the head coach to effectively deliver the intervention. Finally, the mini-basketball intervention program has been successfully implemented and has yielded positive results, as evidenced in our three previous studies [18, 20, 34]. The MBTP was organized into three stages, each incorporating specific tasks designed to meet the distinctive conditions of participants, as outlined in Fig. 2. Comprising four sections—introduction, warm-ups exercises, the intervention itself, and relaxation—the entire session spanned approximately 40 minutes. Throughout the intervention, the children participated in exercises at a moderate intensity, aiming to maintain a mean heart rate between 128 and 148 beats per minute, monitored using the POLAR M430 heart rate monitor. The intervention occurred five times per week, from Monday through Friday, adhering to a consistent schedule, at a designated location, and with the same instructor, spanning a duration of 12 weeks.

### Behavioral outcome measures

Previous studies have shown that several factors, including the severity of ASD, physical fitness, sleep habits, and eating behaviors, influence the social abilities in patients with ASD [35–37]. Thus, we collected essential demographic information about the participants, including age and gender, at the beginning of the study [38, 39]. Simultaneously, we evaluated the physical fitness of preschoolers with ASD using the Chinese Physical Fitness Manual, where the Body Mass Index (BMI) was calculated as weight (kg)/height (m^2^). Speed-agility was assessed through the 2×10m round running test, muscular strength through the standing jump test, and flexibility by measuring forward flexion while sitting. Detailed testing procedures can be found in the published article [34]. To assess symptoms, sleep patterns, and eating habits, we employed the Childhood Autism Rating Scale (CARS) [40], the Children's Sleep Habits Questionnaire (CSHQ) [41], and the Child Eating Behavior Questionnaire (CEBQ) [42]. Our objective was to address any initial discrepancies between the two groups. The CARS scale was assessed and measured by physicians at the hospital. The CHSQ and CEBQ were completed by guardians or teachers of the participants.

Participants in this study were assessed using the Social Responsiveness Scales-Second Edition (SRS-2) to measure the severity of their SCI [43]. This assessment tool has exhibited high internal consistency and retest reliability [44]. The SRS-2 is composed of 65 items grouped into five subscales: social awareness, social cognition, social communication, social motivation, and autistic mannerisms. A higher score indicates a more severe SCI. The person who completed the scale acted as the guardian of the participants, and their role remained consistent both before and after the experimental intervention.

### Rs-fMRI data acquisition

Data and image acquisitions were conducted before and after the experiment using a 3.0T MRI scanner (model: GE Discovery MR750W 3.0T) at the MRI scanning facility at the Affiliated Hospital of Yangzhou University. To ensure the successful completion of the MRI scan for each subject, sedation was administered due to the extended scanning time and the noisy operation of the machine. The sedation management procedure involved instructing the guardian of the autistic child, one day prior to the scan, to keep the participants awake late at night and wake them up early in the morning. Additionally, each subject received an enema containing 10% chloral hydrate at a dose of 0.3 ml/kg (30 mg/kg) every 6 to 8 hours, with the maximum dosage not exceeding 10 ml. In cases where the participant did not respond to mild pain stimulation, a medical professional positioned them in a supine position on the scanning bed. The parameters for the T1-MPRAGE structural scan were set as follows: TR/TE = 7.2/3.1ms, TI = 450ms, slice thickness = 1mm, flip angle = 12°, acquisition matrix = 256 × 256, FOV = 256 × 256mm. The resting-state MRI scanning parameters (EPI scan) were also recorded: TR/TE-2000/30ms, slice number = 28, slice thickness = 4.0mm, pitch = 1mm, flip angle = 90°, acquisition matrix = 64 × 64, FOV = 224 × 224mm.

### Rs-fMRI data preprocessing

Rs-fMRI data underwent preprocessing using the DPARSF software tool (http://www.rfmri.org/dparsf_v2_0). To mitigate potential noise interference, the initial 10 time points of each participant's rs-fMRI data were excluded, leaving the remaining 230 time points for subsequent pre-processing stages, including time correction, correction for head motion, and space standardization. Head movement correction generated horizontal and rotational head movement maps for each subject in the time series. Subjects with significant head translational movement or more than 3° of rotational movement were removed from the data analysis. The spatial standardization employed the DARTEL standard template.

ReHo, developed by Zang Yufeng and colleagues [24], serves as a method for analyzing rs-fMRI data, relying on the Kendall Harmony Coefficient (KCC) for its calculations. ReHo detects neural activity by analyzing the similarity of Blood Oxygen Level-Dependent (BOLD) signal fluctuations in multiple nearby voxels within the same time series [24]. DPARSF filtered the spatially normalized images (0.01-0.08 Hz) and removed linear drift. The KCC values were calculated for each voxel in the entire brain, creating a ReHo map for every subject. This value was divided by the mean KCC value of all voxels in the entire brain to obtain a standardized ReHo map. Subsequently, the standardized ReHo map underwent Gaussian smoothing with a full width at half maximum (FWHM) of 6mm. The impacts of a 12-week MBTP on the ReHo map before and after the intervention were analyzed using the random effects model of flexible factorial design in SPM12. Resulting alterations in ReHo were then extracted. Regions of interest were identified as brain areas showing significant alterations in ReHo. The ReHo value in each region of interest was extracted post-exercise intervention.

### Statistical analysis

Initially, the normality of the distribution for all demographic and behavioral variables will be assessed using the Shapiro-Wilk test. Subsequently, based on the results of the normality checks, we will use SPSS 24.0 software to conduct either an independent samples *t*-test or a Mann-Whitney *U* test to assess the homogeneity of demographic characteristics (BMI and age), and CARS, CSHQ, CEBQ, and physical health development between the two participant groups. This aimed to identify any differences in baseline demographic and behavioral characteristics. Additionally, based on the results of the normality checks, a repeated measures analysis of variance or a two-factor nonparametric analysis of variance was employed to examine the impact of a 12-week MBTP on SRS-2 scale scores in children with ASD. The partial eta-squared (*η*_*p*_^*2*^) value served to indicate effect size. In the event of a significant time × group interaction, a subsequent simple effect analysis would be conducted. Finally, Pearson correlation analysis was utilized to investigate the correlation between alterations in each region of interest (baseline-posttest) and changes in SRS-2 scores (baseline-posttest) after the intervention. This analysis sought to unveil the neural mechanism underpinning the effectiveness of the 12-week mini-basketball intervention in improving SCI in preschool children with ASD. *P*-values < 0.05 were statistically significant.

## Results

### Demographic characteristics

Independent sample *t*-tests and Mann-Whitney *U* test were employed to compare age, physical fitness, CARS, CSHQ, and CEBQ between the two groups. The independent sample* t*-tests results revealed no statistical differences between the groups for BMI (*t *_*(28)*_ = -0.425, *p* > 0.05), flexibility (*t *_*(28)*_ = -1.207, *p* > 0.05), CARS (*t *_*(28)*_ = 0.305, *p* > 0.05), *p* > 0.05), and CEBQ (*t *_*(28)*_ = -0.178, *p* > 0.05). As age, speed-agility, muscle strength, and CSHQ did not follow a normal distribution, the Mann-Whitney *U* test was used to compare the two groups. The results of the Mann-Whitney *U* test showed no significant differences between the groups in terms of age, speed-agility, muscle strength, and CSHQ (all *p* > 0.05). The observed differences were not statistically significant, indicating homogeneity between the two groups concerning demographic indicators, physical fitness status, sleep, and diet (Table 1).

**Table 1:** Demographic characteristics of the two groups of preschool children with ASD (x̄ ± SD)

Variable	Control Group	MBTP Group
Number	15	15
Gender (boys/girls)	13/2	13/2
Age (years)	4.67 ± 0.70	5.17 ± 0.72
BMI (kg/m^2^)	15.88 ± 1.80	15.64 ± 1.29
CARS ^a^	39.80 ± 5.24	40.53 ± 7.70
CSHQ ^b^	58.60 ± 12.29	56.60 ± 4.90
CEBQ ^c^	54.40 ± 20.05	53.40 ± 8.30
Speed-agility (s)	11.83 ± 2.23	13.41 ± 4.10
Muscle strength (cm)	39.67 ± 19.30	48.07 ± 21.10
Flexibility (cm)	19.61 ± 2.43	17.59 ± 6.01

x̄ Mean. *SD* Standard deviation

^a^*CARS* Childhood Autism Rating Scale

^b^*CSHQ* Children’s Sleep Habits Questionnaire

^c^*CEBQ* Child Eating Behavior Questionnaire

### SCI before and after the intervention

The SCI behavioral variables were found to be normally distributed based on Shapiro-Wilk testing. Consequently, a repeated measures ANOVA was conducted to evaluate the effects of a mini-basketball intervention on the total SRS-2 score and the scores of five sub-dimensions in participates. The study revealed a statistically significant time × group interaction in the total score of the SRS-2 (*F*_*(1,28)*_ = 14.514, *p* < 0.001, *η*_*p*_^*2*^ = 0.341), and in the scores of social cognition (*F*_*(1,28)*_ = 15.620, *p* < 0.001, *η*_*p*_^*2*^ = 0.358), social communication (*F*_*(1,28)*_ = 12.460, *p* < 0.01, *η*_*p*_^*2*^ = 0.308), and autistic mannerisms (*F*_*(1,28)*_ = 9.970, *p* < 0.01, *η*_*p*_^*2*^ = 0.263). Accordingly, we performed further simple effect analyses on the four outcomes mentioned above. The results indicated that there were no statistical differences in the baseline scores of the total SRS-2 score and the scores of the subscales between the experimental and control groups (*p* > 0.05). Our findings also revealed significant decreases in the scores of the experimental group on the SRS-2 total score, as well as its social communication and social cognition subscales (*p* < 0.05). Conversely, the control group exhibited a significant increase in scores on the SRS-2 total score, social communication, and autistic mannerisms subscales (*p* < 0.05). These findings strongly indicate that the MBTP could improve SCI of participates, particularly in the domains of social communication, social cognition, and autistic mannerisms.

**Fig. 3:**
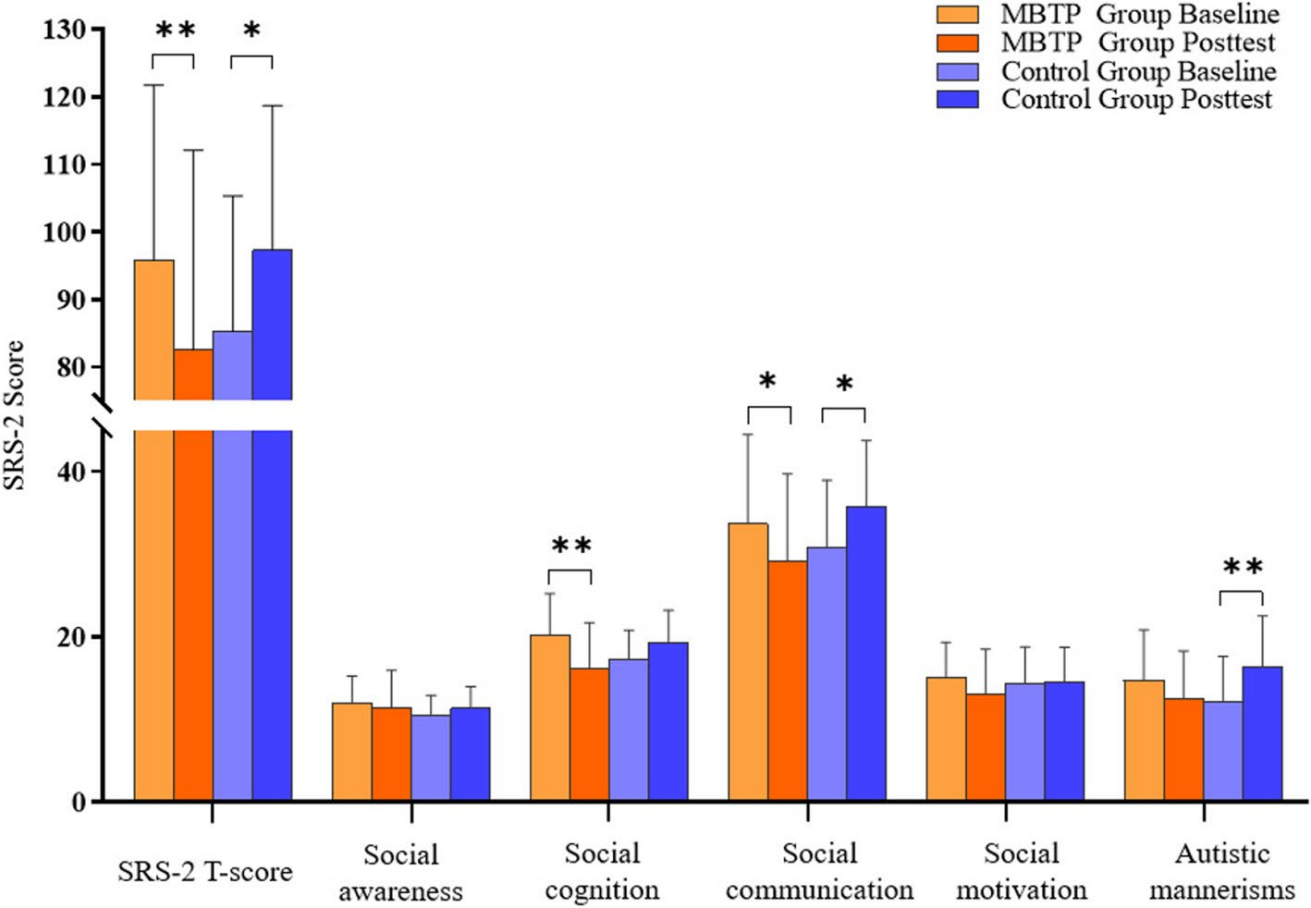
Changes in SCI in preschool children with ASD before and after intervention. Notes: Alterations in the total score of the SRS-2 and its five subdimensions in preschool children with ASD before and after intervention. Error bars represent standard deviation. * *p* < 0.05; ** *p* < 0.01

No statistical difference was found in the scores for the social awareness subscale (*F *_*(1,28)*_ = 3.416, *p* > 0.05, *η*_*p*_^*2*^ = 0.109) and social motivation subscale (*F *_*(1,28)*_ = 2.450, *p >* 0.05, *η*_*p*_^*2*^ = 0.080) in the time × group interaction, suggesting that the mini-basketball exercise intervention did not result in improvements in social motivation and perception (Table 2 and Fig. 3).

**Table 2:** Alterations of SCI in preschool children with ASD before and after intervention (x̄ ± SD)

Variable	MBTP Group		Control Group		*F*	*η*_*p*_^*2*^
	Baseline	Posttest	Baseline	Posttest		
Social awareness	12.1 ± 3.20	11.5 ± 4.47	10.5 ± 2.45	11.4 ± 2.59	3.416	0.109
Social cognition	20.2 ± 5.06	16.3 ± 5.46	17.3 ± 3.56	19.3 ± 3.89	15.620^***^	0.358
Social communication	33.7 ± 10.79	29.1 ± 10.06	30.9 ± 8.06	35.7 ± 8.09	12.460^**^	0.308
Social motivation	15.1 ± 4.17	13.1 ± 5.42	14.4 ± 4.39	14.5 ± 4.19	2.451	0.080
Autistic mannerisms	14.7 ± 6.12	12.6 ± 5.69	12.3 ± 5.36	16.4 ± 6.15	9.970^**^	0.263
SRS-2 T-score^a^	95.8 ± 25.86	82.5 ± 29.55	85.3 ± 20.04	97.3 ± 21.35	14.514^***^	0.341

*x̄* Mean, *SD* Standard deviation. Numbers presented are *F* statistics showing the tests of the interaction effect of group and time. *η*_*p*_^*2*^: Partial eta-squared represents the effect size

^a^The total score of the Social Responsiveness Scale Second Edition (SRS-2). ** *p* < 0.01. *** *p* < 0.001

### ReHo before and after the intervention

**Fig. 4:**
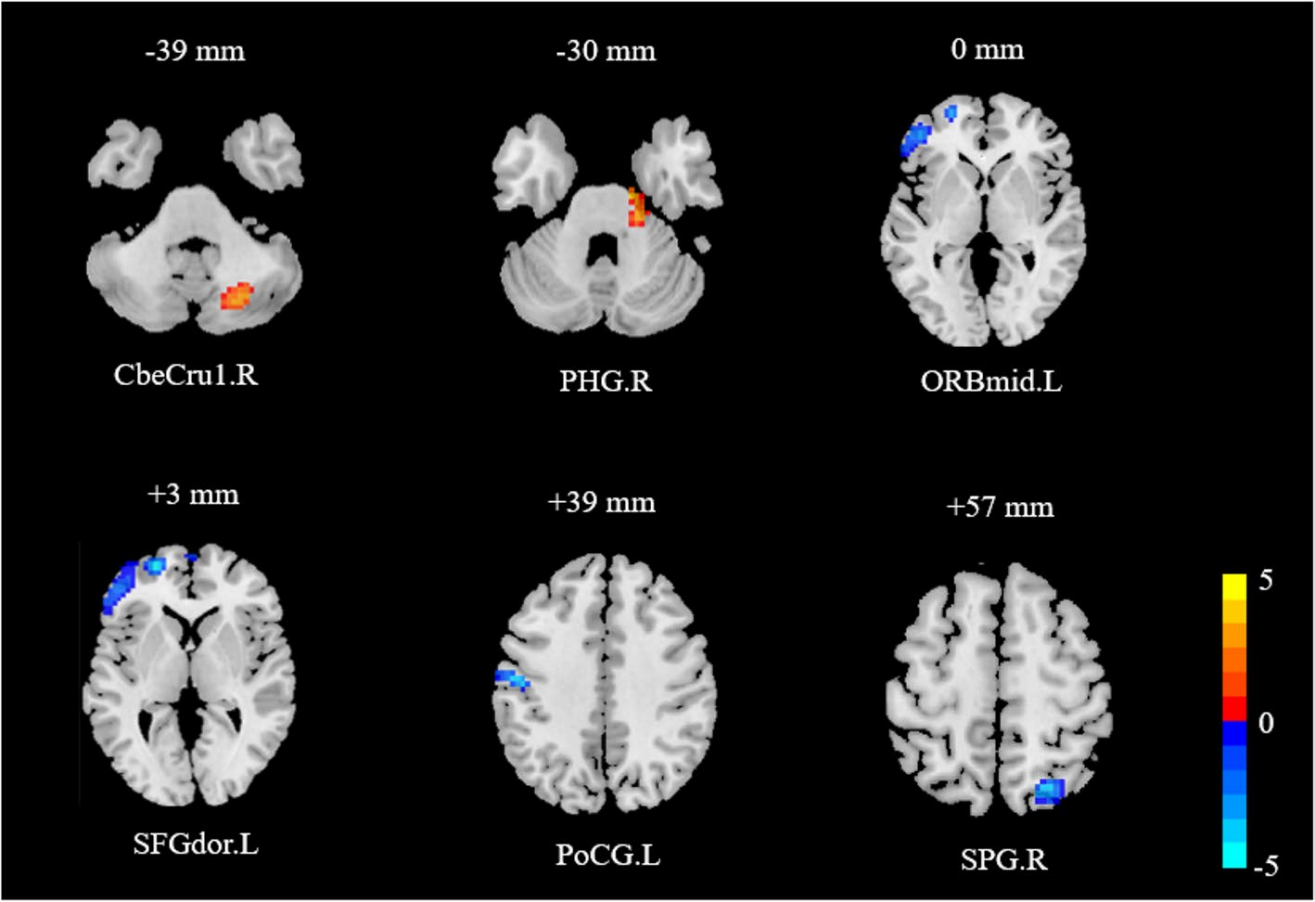
Alterations in ReHo values for preschool children with ASD before and after the intervention. Note: The figure displays the Z-axis coordinates while the luminance bands and numbers on the lower right side indicate the correlation between luminance and *t* values in the brain regions. Red areas signify a significant increase in ReHo values, while blue areas signify a significant decrease in ReHo values. CbeCru1.R: right Cerebelum_Crus1. PHG.R: right parahippocampal. ORBmid.L: left middle frontal gyrus, orbital part. SFGdor.L: left superior frontal gyrus, dorsolateral. PoCG.L: left postcentral gyrus. SPG.R: right superior parietal gyrus. The statistical significance level was defined as* p* < 0.01, uncorrected, with a minimum cluster size of 50

The ReHo images of both groups of participants were analyzed before and after the test using the flexible factorial design of SPM12, examining alterations in ReHo images before and after the intervention. Significant differences were observed in resting-state ReHo values of whole-brain BOLD signals before and after intervention in participates. Specifically, ReHo values increased significantly in the right Cerebelum_Crus1 (CbeCru1.R, Peak MNI coordinate: 24, -69, -39; 19 voxels, *t* = 4.24) and the right parahippocampal (PHG.R, Peak MNI coordinate: 15, -15, -30; 12 voxels, *t* = 4.41). Conversely, ReHo values significantly decreased in the left middle frontal gyrus, orbital part (ORBmid.L, Peak MNI coordinate: -45, 45, 0; 26 voxels, *t* = -4.05), the left superior frontal gyrus, dorsolateral (SFGdor.L, Peak MNI coordinate: -24, 57, 3; 71 voxels, *t* = -5.10), the left postcentral gyrus (PoCG.L, Peak MNI coordinate: -48, -15, 39; 58 voxels, *t* = -4.64), and the right superior parietal gyrus (SPG.R, Peak MNI coordinate: 24, -66, 57; 56 voxels, *t* = -4.54) (Fig. 4).

### Correlations between SCI changes and ReHo alterations

**Fig. 5:**
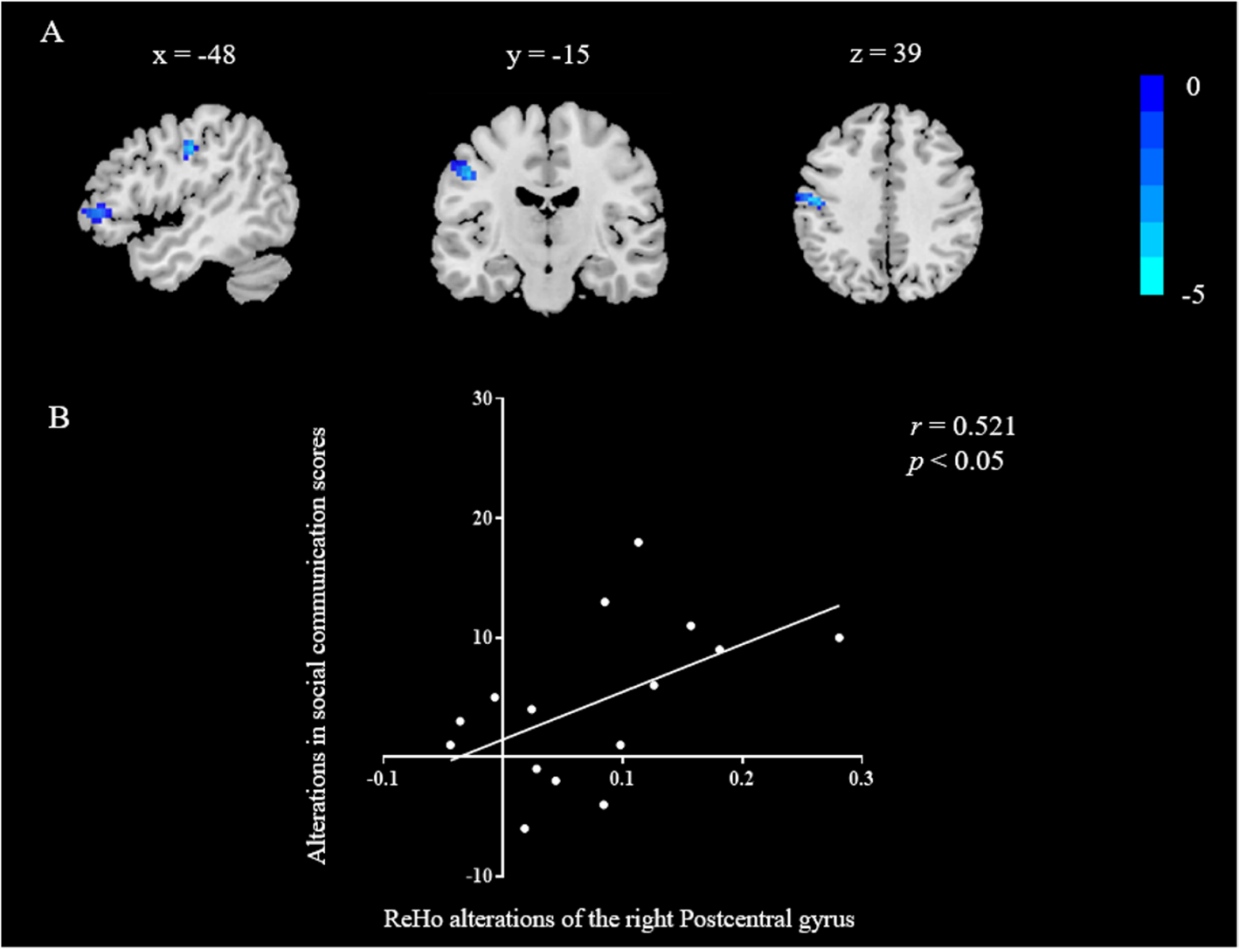
Correlation between the enhancement of social communication ability and the alteration of ReHe in the left postcentral gyrus. Note: A. The blue area indicates a significant decrease in the ReHo value in the left postcentral gyrus. Figure coordinates correspond to the x, y, and z directions. B. The alteration of ReHo in the left postcentral gyrus was positively associated with the change of social communication score in preschool children with ASD after the intervention. Where the abscissa is the change in the PoCG.L ReHo values, and the ordinate is the change of the social communication scores

The brain regions that displayed significant alterations in ReHo values before and after the 12-week intervention were identified as regions of interest. Subsequently, ReHo alterations within these regions were extracted for all subjects before and after the intervention. The analysis aimed to assess the correlation between ReHo changes (baseline-posttest) and the corresponding change in SCI behavior scores (baseline-posttest). The study revealed a significant correlation between the decrease in ReHo values in the PoCG.L and the change in social communication scores (*r* = 0.521, *p* < 0.05). Nevertheless, no significant correlation was found between alterations in the ReHo values of the right Cerebelum_Crus1, right parahippocampal, left middle frontal gyrus, orbital part, left superior frontal gyrus, dorsolateral and right superior parietal gyrus, and the total score of SRS-2, or changes in behavioral performance within its subdimensions (Fig. 5).

## Discussion

The paper explored the effects of a 12 weeks MBTP on SCI and ReHo in patients with ASD using rs-fMRI technology. The findings indicated that after mini-basketball intervention, the experimental group had significantly lower scores on the SRS-2 total score and its subscales for social cognition, social communication, and autistic mannerisms compared to the control group. Besides, the experimental group showed increased ReHo values in the CbeCru1.R and PHG.R, and decreased ReHo values in the ORBmid.L, SFGdor.L, PoCG.L, and SPG.R. Furthermore, the decrease in ReHo in the PoCG.L was positively associated with the alteration in social communication scores.

We observed a significant improvement in SCI among preschool children with ASD following a 12weeks MBTP. This outcome aligns with findings from prior studies and interventions addressing SCI in children with ASD [18, 45]. Notably, our intervention had a significant positive impact on social cognition, social communication, and autistic mannerisms in these children. Previous research has shown that various movement-based interventions, such as yoga [46], martial arts [47], and judo [48], could effectively enhance social abilities and behaviors in children diagnosed with ASD. These interventions, centered around general movement, encourage participation in group activities and games, fostering self-expression, social communication, and creativity [49]. Therefore, we hypothesized that an MBTP incorporating various social training components, such as greeting to peers, parents, and teachers, as well as engaging in passing and catching drills with peers, could significantly enhance social communication abilities in individuals with ASD. Nevertheless, the MBTP did not significantly improve social awareness and social motivation in individuals with ASD. In contrast, a prior study on a 12-week equestrian exercise intervention found a significant enhancement in social motivation among in individuals with ASD [50]. This discrepancy might be attributed to the fact that our MBTP primarily focused on stimulating sensory responses and teaching technical movement and other aspects rather than specifically targeting social motivation [51]. Additionally, a meta-analysis indicated that interventions with a duration of 12 weeks or more, a frequency of 3 or more times per week, and a duration of 90 or more minutes each time yielded the most significant improvement in core symptoms among individuals with ASD [10]. In contrast, our MBTP faced limitations regarding the intervention duration. Therefore, it is highly conceivable that the variation in the impact of the intervention may have been influenced by the dosage used in the intervention.

We observed increased ReHo values in the CbeCru1.R and PHG.R, coupled with reduced ReHo values in the ORBmid.L, SFGdor.L, PoCG.L, and SPG.R in the ASD group, consistent with our hypothesis and previous research. It has been reported that the human brain can exhibit changes in plasticity through exercise, learning, and various experiences, particularly in elderly individuals and children, offering opportunities for enhancing brain development through exercise interventions [3, 52]. The effect is more common in elderly individuals and children, demonstrating that the plasticity of the human brain offers opportunities for enhancing brain development through exercise interventions [53]. Beyond their high-level cognitive, sensorimotor, and social functions, the cerebellum and parahippocampal gyrus play essential roles in the pathophysiology of ASD [54, 55]. Individuals with ASD often exhibit abnormal brain activity in these regions, as evidenced by reduced local brain activity in the bilateral cerebellum_Crus1 and parahippocampal gyrus compared to TD children and adolescents, as observed through rs-fMRI technology [56–58]. Similarly, disruptions in regional connectivity have been noted in individuals with ASD compared to TD children at various ages, particularly during early childhood, with a significant reductions in ReHo values observed in the cerebellar lobule and other brain regions [59]. The well-established significance of the frontal and parietal lobes in processing social information, episodic memory, and social cognition is supported by existing literature [60–62]. Abnormalities in these regions have been identified in individuals with ASD, including higher ReHo values in the frontal and parietal lobes compared to TD children and adults [25, 59, 63–65]. These abnormal brain activities may be associated with the pathophysiological processes of ASD, as indicated by studies characterizing functional brain development in children with ASD. We postulated that damage to the cerebellum, parahippocampal gyrus, frontal lobe, and parietal lobe in individuals with ASD led to aberrant brain activation patterns. Furthermore, we hypothesized that 12 weeks of mini-basketball training altered the plasticity of these brain regions in preschool children with ASD, resulting in changes observed in CbeCru1.R, PHG.R, frontal lobe (primarily in ORBmid.L and SFGdor.L), and parietal lobe (primarily in PoCG.L and SPG.R) with altered activation patterns and ReHo values.

Furthermore, we found a significant positive correlation between changes in social communication scores and reductions in ReHo values in the left postcentral gyrus within the parietal lobe in preschool children with ASD, suggesting that improvements in social communication skills are linked to reductions in ReHo values in the left postcentral gyrus. Existing studies have consistently indicated that patients with ASD often exhibit local hyperconnectivity in the parietal lobe [64]. Cross-sectional studies have further established that abnormal connectivity in parietal regions is linked to social, motor, and attentional deficits in individuals with ASD [66, 67]. The postcentral gyrus, situated in the parietal lobe, houses the primary somatosensory cortex involved in perceiving and analyzing sensory information. Researchers have noted a strong correlation between sensory problems resulting from abnormal activation of the postcentral gyrus and the core symptoms of patients with ASD [68, 69]. As a result, we speculated that damage to the postcentral gyrus might be associated with SCI in individuals with ASD. In summary, there is mounting evidence indicating a strong link between themajor symptoms of individuals with ASD and damage to the postcentral gyrus. Building on the analysis of prior research and our own study's findings, it is highly conceivable that a 12-week mini-basketball exercise can enhance the development of social abilities in preschool children with ASD by remodeling brain function. This hypothesis gains support from existing evidence. Chen et al. [31] explored the neural mechanisms through which aerobic exercise enhances executive function in children. Their research indicates that physical exercise holds the potential to improve brain plasticity and foster the development of executive function in children by enhancing ReHo values. Consequently, we hypothesized that MBTP could ameliorate the social abilities in children with ASD by reducing ReHo values in the postcentral gyrus within the parietal lobe. This research illuminates the neural mechanism underpinning the impacts of physical exercise on SCI in preschool-aged children with ASD, particularly from the perspective of ReHo. It contributes to an expanded understanding among researchers of the neural mechanisms related to SCI in children with ASD.

## Limitation

This study has several limitations. Firstly, while MBTP demonstrated a positive impact on SCI in preschool children with ASD, no significant improvements were observed in social motivation and social awareness. Future research should focus on optimizing the MBTP program to enhance the overall SCI of children with ASD. Secondly, the SRS-2 was completed by the participants' guardians. Although the SRS-2 exhibits good reliability and validity, it incorporates subjective elements. It is essential to recognize this aspect when interpreting the results. Thirdly, the statistical analysis results in the random effects model analysis were uncorrected. Therefore, caution is advised in interpreting the findings that mini-basketball practice improves SCI and ReHo values in preschool children with ASD. Finally, despite the similarities between our study results and those of previous research, differences exist. Factors contributing to these variations may include the limited sample size, with a significant number of dropouts during the study due to incomplete posttest assessments and other reasons. Additionally, different sample characteristics, such as intelligence quotient, age, and symptom severity, may have played a role. Future studies should address these limitations by increasing the sample size and conducting larger-scale investigations to confirm these findings.

## Conclusion

Overall, this study offers valuable insights into the relationship between MBTP and SCI in preschool children with ASD. The positive effects of MBTP on SCI were substantiated through a comprehensive analysis of both behavioral and neuroimaging data. Moreover, we identified a neural mechanism underpinning these improvements, specifically noting a reduction in ReHo values in the left postcentral gyrus linked to an enhancement in social communication abilities. These results contribute substantially to our understanding of the potential impact of MBTP in alleviating SCI in preschool-aged children with ASD. These findings establish a foundational basis for considering the integration of MBTP into educational and medical interventions, highlighting its potential as a valuable component in addressing SCI among this specific population.

## Data Availability

The anonymized dataset used for analysis will be made available from the corresponding author upon reasonable request.
